# A New Treatment of Parturient Paresis of Cows

**Published:** 1898-09

**Authors:** Olof Schwarzkopf

**Affiliations:** Flushing, N. Y.


### A NEW TREATMENT OF PARTURIENT PARESIS OF COWS.

By Olof Schwarzkopf, V.M.,
FLUSHING, N. Y.

Of the diseases of domestic animals incidental to seasons, none is of a more serious nature and more disappointing to the veterinarian with rural practice than the so-called milk-fever of cows. Every year this disease makes its appearance at the calving season, and continues more or less unabated during the hot summer months. From the grave character of the disease ; from the fact that it befalls only valuable, well-bred and well-fed cows; from the conflicting theories of the pathology of the disease, and the utter absence of an accurate and controllable therapeutics, it remains a disease dreaded by all veterinarians whose lot it is to deal with it in practice.

[1] Read at the annual meeting of the New York State Veterinary Medical Society, New York, September 15, 1898.

The theories on the pathology of milk-fever, historic and present, are interesting not only in themselves, but specially so as illustrating the periodic changes in medical ideas. They can, however, only briefly be referred to, as the object of this paper is to be concise and practically instructive. The oldest pathological theory which may have a claim to be scientific is that of Bentele, dating back to the early years of this century. He and his followers considered the disease a lacteal metastasis, and in old veterinary books we read of milky contents in the kidneys, of milky urine, of lactiform substances found in the lungs, etc. This theory has been forgotten by the present generation of veterinarians, but if signs are true it may be revived in a modified form. Besides the later theories of English writers which are known to you, there is that of Harms, of Hanover, who explained it as a result of suction of air into the bloodvessels during parturition, followed by cerebral anæmia and paresis. This hypothesis was born at a time when fatal results were obtained by the absorption of air during operations at the early days of modern surgery, and it was apparently substantiated by the presence of air-globules in cerebral vessels, a condition which later researches have shown to be the result of postmortem blood-decomposition. Then there is the theory of Frank, of Munich, who considered the disease a result of the sudden and grave circulatory changes produced by the abrupt loss of a large area of blood-circulation in the uterus, forcing large quantities of blood into new channels, especially the brain, thus producing cerebral congestion, followed by encephalic œdema and anæmia. This complex theory, based on an intimate anatomical study of fœtal and uterinal circulation, withstood the scientific criticism of years, and was until recently accepted all over the Continent of Europe. But of late it has been more or less superseded by the more modern theory of Schmidt-Mulheim, who advanced the theory that the disease was due to auto-intoxication by certain cadaveric alkaloids similar to the sausage-poison. He argued that these poisons develop within the uterus by the decomposition of the lochia, favored by the rapid occlusion of the neck of the uterus as observed in milk-fever. He based his theory on the result of the researches in experimental poisonings of Hoppe-Seiler, who discovered that a number of alkaloids, especially ptomatropin, ptomacurarin, and mytilotoxin, produce a paralysis of certain muscular groups, such as the pharynx, eye, etc., and in severe cases end in a paresis of the striated muscular fibres of the extremities and the unstriated fibres of the intestine and bloodvessels. No one can deny that these symptoms have a striking resemblance to those so commonly exhibited by milk-fever. This brief review of the principal theories shows that they have ever been influenced by the predominant thoughts and discoveries in pathology, and they demonstrate the intense desire of our professional predecessors to unravel the mysteries of the nature of this disease.

If we turn to the therapy of milk-fever it is impossible to enumerate all the drugs that have been applied and found wanting. They number by the hundred; they range from the most crude and empirical applications of large quantities of drugs to the delicate use of the new biological products, and they have all been found worthless or of doubtful value. True, there are practitioners who believe they have found a "sure cure;" but sooner or later they will find, as we all have found, that at one time we may be quite lucky with a certain kind of treatment, while the second or third time we are decidedly unlucky. It is, as Professor Viborg once said: "Manchmal hilft alles, Manchmal hilft nichts" (Sometimes anything helps, sometimes nothing helps). I may summarize these points by saying that the medicinal treatment of this disease demonstrates most truthfully to the unbiased observer that it is not the drugs that act upon the diseased body, but that it is the living organism that reacts upon the drugs as long as it is capable of response. If from one cause or another the life of the animal is stunned, the so-called physiological action of drugs is left to be ampty phrase.

Nevertheless, there are certain methods of external and internal application which, if employed at the proper time and with proper precautions, tend to assist the struggling organism to overcome its morbid condition. They should never be dispensed with, whatever our future treatment may be. The first indication to follow is to put the animal in a comfortable position on a copious and clean bedding, which is not always an easy matter. Then we must attend to the abnormal external temperature of the body by the use of external stimulants, assisted by friction with brushes, and by covering the body in woollen blankets. Following this the udder should be thoroughly milked out, while we empty the rectum by manual extraction of the feces, injecting immediately afterward a pint of a mixture of glycerin and water of equal parts, which rarely fails to assist in periodic evacuation of ingesta. Quite often I have emptied the bladder with apparent relief.

But when we now attempt a treatment of the alarming symptoms of distress so commonly exhibited by the animal, the deep mental depressions, the paralysis of the muscles, and the complete cessation of peristaltic movement, we fail. As the pharynx is early paralyzed, deglutition is difficult if not impossible, and excludes the application of medicines *per os.* Thus we must rely upon hypodermic medication. To counteract the nervous depression veratrine, caffeine, strychnine, spirits of camphor, etc., have been recommended. I have applied them all, and found that veratrine is the only agent to which the organism regularly responds by a profuse perspiration, but from which no lasting benefit is visible. To stimulate the peristaltic movements, eserine has given me prompt results, but in severe cases it is absolutely inefficient. Of the more modern methods of treatment the use of the normal salt solution has given me promising results. Out of three cases so treated two recovered. The solution undoubtedly produces a general reviving effect, easily perceptible. But a relapse may follow which leaves the animal in a worse condition, and repeated infusions of such large quantities into the jugular vein of cows so diseased are impracticable because the muscles of the bent neck, if straightened, contract spasmodically, preventing the operation. Besides, the cow-stable cannot readily be fitted into a laboratory, and the preparation of the solution and the process of infusion require a cleanliness and an assistance that cannot be had in such surroundings. To overcome these difficulties I have tried the hypodermic injection of the artificial serum of Cheron, which was recommended as a substitute for the normal salt solution as being of more precise action and applicable in smaller quantities. Of two cases so treated one came to rapid recovery, the other ended in death, which appeared to be hastened by the injections.

This was my experience with the treatment of the diesase when I read, last winter, a short notice in the *Berliner Thierartzliche Wochenschrift* announcing the discovery of a new treatment of milk-fever by Veterinarian Schmidt-Kolding. According to him, the cause of the disease must be located in the udder which, by the suddenly increased lactation after birth, loosens great masses of old glandular cells (colostrum) in a sort of cleaning process. These undergo a decomposition, and form toxins, which are absorbed into the blood-circulation, resulting in auto-intoxication. He therefore directed his therapy against the udder, and particularly against an abnormally high milk-secretion, both qualitative and quantitative. It being known that iodide of potassium has the effect of decreasing the milk-secretion, he experimented with a solution of this drug by infusing it directly into the milk-glands. Out of fifty cases so treated, forty-six recovered.

Schmidt's treatment is in detail as follows: After attention to the first symptomatic treatment the udder is milked out, thoroughly cleaned with soap and water, and the udder and teats disinfected with a lysol solution. Iodide of potassium, 7–10 grammes, is then dissolved in one litre of freshly-boiled water, the solution filtered and cooled to 40°, and slowly infused into the four teats, equal parts. The infusion is accompanied by massage of the udder. The apparatus for infusion consists of a glass funnel, rubber-tube of 1½ metre length, and a milking-tube. He recommends to give 5 grammes of caffeine subcutaneously if the pulse is weak, and if deglutition is not impaired an aloe-powder is given *per os.*

This treatment is certainly a wide departure from our customary methods. It is interesting because it is new, simple, and direct, and if the hypothesis of the cause of the disease is correct it must be scientific. I confess that I was skeptical at first, but was resolved to try and criticise afterward. Thus I can report the following cases:

CASE I. *Failure.*—On May 23d, 5 P.M., I received a hurry-call to come to a cow near Whitestone, which was bloated. When I arrived I found the animal, a Jersey, lying in a pasture, the head bent to the left side. She was in great distress, groaning heavily, grinding the teeth, and was in tympanitic condition. From a hurried verbal examination of the owner, which brought out the fact that the animal had calved two days previously, I informed him that the cow had milk-fever in a severe form, and that the prognosis was unfavorable. I resolved to try Schmidt's treatment, and, after giving directions for first treatment, drove to the nearest drug-store to procure iodide of potassium and a new syringe, as I had not yet acquired an apparatus for infusion. When I returned I found the cow in worse condition, but proceeded to prepare the iodide solution in a nearby kitchen. I found the injection into the teats a tedious process, although I had no difficulty in inserting the syringe, but it had become dark and I had to proceed by the light of a lantern. When I had finished and given a hypodermic injection of eserine, I watched the animal for two hours, but could see no effect of the treatment. When I called early next morning I was informed that the cow had died at 4 A.M. I opened the udder and found that the solution had been entirely absorbed and that no inflammation had resulted from the manipulations.

CASE II. *Recovery.*—On June 2d, 10 A.M., I was called to College Point to a cow reported apparently suffering with milk-fever. I found the cow, a Jersey, in a pasture, unable to rise and trying to keep the head straight, which would occasionally fall to the left side with a jerk. The case presented a light form of the disease, but I made the prognosis doubtful. I was prepared for Schmidt's treatment; made the solution in a restaurant, and injected it through the apparatus, which worked like a charm. I applied injection of 10 grammes of caffeine, and, noticing that the animal was bellowing for her calf, which had been removed by the owner, I had it returned and bedded next to her, which quieted her greatly. At 7 P. M. I received a telephone message that the cow was up, but very weak. I called the next morning, and found her still weak and swaying from one side to another when moved. I applied a stimulating drink and left. On the third morning the message said the cow was still weak, but otherwise all right.

CASE III. *Recovery.*—On July 6th, early in the morning, I received a call to come to Douglaston to a cow that had calved and was sick. I found the animal, a fine Jersey, in a box-stall, with all the symptoms of a severe form of milk-fever. I informed the owner of the unfavorable prognosis and then applied Schmidt's treatment at 9 A.M., injecting 10 grammes of the iodide solution. Being requested to call again, I arrived there at 5 P.M., and found the cow little if anything changed, but not worse. I resolved to give a second injection of the iodide solution, reduced to 5 grammes, which was easily done. I called again the next morning and found the cow still lying down, but head erect and trying eagerly to consume a bran-mash. Third morning the cow was reported still lying, but otherwise doing well. Fourth morning cow reported up and apparently well.

CASE IV. *Recovery.*—On July 14th, 4 P.M., I was passing through Whitestone, when I was halted and requested to attend to a sick cow. I found same in pasture, suffering from a light attack of milk-fever. After applying first treatment I went home to get my infusion-apparatus, and one hour later I was applying Schmidt's treatment. I called the next morning and found the cow still lying, but being coaxed with her calf she got up with assistance. She remained weak for two days.

In conclusion, I wish to say that favorable as these results appear to be, I have learned to be cautious, and I shall warn you against too much enthusiasm. True, I have never had three cases in succession that recovered as well as those above cited; but we all have seen good recoveries before. One point I want to make stand out clearly, namely, that Schmidt's treatment requires a painstaking thoroughness and cleanliness, and that the manipulations of infusion are a delicate process that should not be performed by crude hands. If you will observe these points scrupulously and skilfully, then the treatment will be a pleasure to yourselves and give great satisfaction to the intelligent class of your clients. I hope you will report your good results and not pass in silence over your failures, for it is rather by such that we learn than by unlimited success.

